# A Contribution to the Curiosities of Medical Experience

**Published:** 1848-11

**Authors:** T. M. Harris

**Affiliations:** Harrisville, Va.


Our supply of editorial matter falling a little short of the reserved space, we select for the vacant niche, the following article from our contemporary, the Western Journal of Medicine and Surgery:

*A Contribution to the Curiosities of Medical Experience.* By T. M. Harris, M. D., of Harrisville, Va.

The novel and remarkable character of the following case constitutes my apology for communicating it to the profession.

On the 19th of May, 1846, I was summoned to attend a young Dutchman in this neighborhood, and received the following history of his case.

He had been suffering from an attack of piles, and having been informed that the disease could be cured by introducing the neck of a well greased bottle containing some hot spirits of turpentine, he undertook to prove the remedy. But unfortunately, using nothing larger than a half pint flask, and having, as I suppose, a more than ordinarily capacious outlet to the alimentary canal, the flask slipped in, and the sphincter closed upon it.

Here is a dilemma—a man with a half pint flask in his rectum seeks relief; and what is to be done? Notwithstanding the case borders a little upon the ridiculous, it became, to me, a subject of most serious and anxious concern. At length, however, I resolved upon a plan, and accordingly went to a blacksmith and had a pair of forceps made somewhat after the fashion of the obstetrical instrument, with blades about seven inches long, by about three-fourths of an inch wide, and handles eight or ten inches long. These being prepared, and the blades well greased, I introduced a blade at a time so as to inclose the bottle, locked the instrument, and commenced my efforts at extraction. But the blunt end, or bottom of the bottle presenting, I soon satisfied myself that it would be no easy task to effect its removal. At length, by the force of my efforts, I smashed the flask in fragments. Having no further use for my forceps, I laid them aside and set myself carefully to work, removing it, a piece at a time, with my fingers. This I completely accomplished after laboring faithfully for about three hours. I then washed the rectum by throwing up large quantities of warm water; ordered a dose of sulph. magnesia, and in three days had the satisfaction of seeing my patient about his employment.

On the 29th of January, 1847, I was called to see the same patient, and informed that a similar mishap had befallen him, the body now introduced being a beet. I made an examination, and could trace with the finger the large end of a beet of such dimensions as to cause the utmost astonishment; and to increase the difficulties of the case, it had been retained more than 48 hours, the patient having entertained the intention of dying like a hero, without disclosing his condition; from which determination however, the intensity of his sufferings forced him to depart.

There was now a good deal of tumefaction and tenderness about the anus; and very great tenderness of the abdomen generally—vomiting had set in. I again introduced my forceps, but with great difficulty, on account of the tumefaction and soreness of the parts, and soon found that I could not make the necessary extractive efforts without having my forceps slip off: the patient was also exceedingly irritable, and could not endure the necessary force. I now took my forceps to the smith, had the width of the blades reduced one-fourth, and the points turned in so as to form a hook, obtained two or three assistants, and returned to the novel operation.

Having premised a free bleeding and the hot bath, so as to obtain a good degree of relaxation, I administered 35 drops of the tinct. opium, and having placed my patient on his knees and strapped him down tightly over some chairs, I again introduced my forceps, and quickly succeeded in bringing away a beet nearly seven inches in length, and in its largest diameter about three and a half inches.

It had evidently been selected by my patient on account of its size, in order that it might be impossible for it to be taken in; and feeling thus secure, he had introduced the small end, and pressed down upon it with his whole weight.

I now administered injections, and laxative doses, and restricted my patient to a low diet for two or three days, when he again resumed his employment.

August 30th, 1848.

